# Methods for the dietary assessment of adult kidney stone formers: a scoping review

**DOI:** 10.1007/s40620-022-01259-3

**Published:** 2022-02-15

**Authors:** Constance Legay, Tropoja Krasniqi, Alice Bourdet, Olivier Bonny, Murielle Bochud

**Affiliations:** 1 Department of Biomedical Sciences, University of Lausanne, Switzer Rue du Bugnon 27, 1011 Lausanne, Switzerland; 2 Service of Nephrology, Lausanne University Hospital, Lausanne, Switzerland; 3 Department of Epidemiology and Health Systems, Unisanté, Lausanne, Switzerland; 4 NCCR Kidney.CH, Zürich, Switzerland

**Keywords:** Kidney stones, Dietary assessment, Nutritional epidemiology, Scoping review

## Abstract

**Background:**

Kidney stones are a frequent and potentially severe condition, affecting 5–10% of the European population. Causes are multifactorial, diet in particular plays a major role in the formation and management of kidney stones. The aim of this scoping review is to assess the methods used to study the diet of adult kidney stone formers.

**Methods:**

We conducted a systematic search in Medline Ovid SP, Embase, Cinahl, Cochrane (CENTRAL), Web of Sciences databases on June 10th, 2020. Self-report methods (such as food frequency questionnaires or 24-h dietary recalls), objective nutritional biomarkers and controlled diets were considered. We analyzed the selected publications based on the origin of participants, study design and dietary assessment methods used.

**Results:**

We screened 871 publications and included 162 of them. Most studies included participants from North America and Europe and were observational. Short and cost-effective tools such as food frequency questionnaires and other questionnaires were the most frequently used. Moreover, food diary was a frequently selected method to study the diet of kidney stone formers. New technologies (e.g. online questionnaires, phone applications, connected tools) were rarely used.

**Conclusion:**

Accurate reporting of the methods used in nutritional studies is of key importance to interpret results and build evidence. Assessing long-term dietary intake is still a challenge for nutritional epidemiology. A combination of self-report methods with objective dietary biomarkers and new technologies probably represents the best way forward.

**Graphical abstract:**

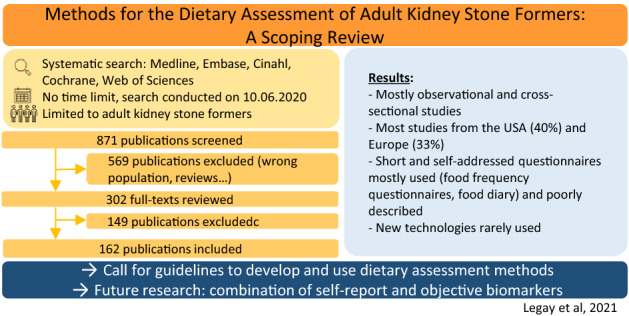

**Supplementary Information:**

The online version contains supplementary material available at 10.1007/s40620-022-01259-3.

## Introduction

Kidney stones are one of the most common diseases of the urinary tract, with a prevalence estimated at 5–10% in Europe [1]. This prevalence has increased in the last decades, with changes in nutritional and lifestyle habits or global warming as possible causes [1, 2]. Many studies have explored the association between diet and kidney stones, establishing dietary risk and protective factors [3–6].

Kidney stones are of great concern for public health because of their associated morbidity and cost [1, 7]. Efficient preventive measures, including dietary recommendations, are thus becoming more and more important [8]. In this context, nutritional studies are of key importance to learn more about the impact of diet on kidney stones.

There are two main categories of dietary assessment methods. First, self-report methods are based on participants’ reports of their dietary consumption. These methods are based on recall (e.g. food frequency questionnaires (FFQs), 24-h dietary recalls) or based on real-time recordings (e.g. food diaries) [9–13]. Second, objective nutritional biomarkers are measured in biological samples such as blood, urine or nails [9–14]. Each method has strengths and limitations and different tools explore different aspects of food consumption [9–13]*.*

The aim of this scoping review [15] is to assess the methods used to study the diet of adult kidney stone formers and provide a better understanding of how researchers conducted nutritional studies. This may help guide further research and improve the quality of evidence in this field [16].

## Methods

The PRISMA-ScR checklist was used for reporting [17].

### Search strategy

We identified key words and prepared search equations specific to a database with the help of a librarian (Thomas Brauchli). We first defined the target population using terms such as “urolithiasis, kidney stone, urine calculi”. We then introduced the concept of dietary assessment with terms such as “nutrition assessment, diet records, eating, fluid consumption”, indicating more specifically methods of interest “24 h recall, food frequency questionnaire, online questionnaire, photo app”. We finally added terms to exclude animal and pediatric studies “not animals, not infant, child”.

A systematic search of Medline Ovid SP, Embase, Cinahl, Cochrane (CENTRAL), Web of Sciences databases was conducted on June 10^th^ 2020 by TB using those search equations. We did not include a time limit and we considered only articles written in English (full equations in Supplementary material).

We added seven publications of interest by “hand-searching” [6, 18–23]. Furthermore, as the search equations did not include metabolomics, we conducted a focus search in PubMed with the terms “metabolomics” and “kidney stones” in January 2021. This search gave 16 results, two publications were selected and added to the review [24, 25].

### Eligibility criteria

We selected publications that studied the diet of adult kidney stone formers. We were specifically interested in the dietary assessment methods and considered self-report methods (such as FFQs or 24-h dietary recalls), objective nutritional biomarkers and controlled diets (participants ingested a known amount of food and fluids) as this is another way of knowing the dietary intake of participants. Moreover, we added terms in the search equations to identify new technologies such as online questionnaires, phone applications or connected tools.

We included only studies in adult (> 18 years old) stone formers. We considered kidney stone formers with associated conditions, such as diabetes or obesity. We excluded studies focusing on struvite stones, as their formation differs significantly from the other stone types. We also excluded comments, editorials or letters.

### Study selection

Two reviewers (AB and CL) did a first selection based on titles and abstracts using the online collaborative platform Rayyan (Rayyan Systems Inc.). When a disagreement occurred, discussion between the two reviewers was usually sufficient to reach a consensus. A third reviewer (OB) helped resolve the situations where agreement could not be reached.

After this first selection, two reviewers (TK and CL) screened the full-texts and extracted data from the publications. The final decision to include a publication was based upon agreement between the two reviewers (TK and CL).

### Data extraction

Data from a publication were extracted by only one reviewer (TK or CL) using a standardized extraction table in Microsoft Office Excel version 2016. The team (OB, MB, TK and CL) discussed the items chosen for the extraction table together. The extraction table was then first tested on a subset of publications and some items were added or clarified. The final extraction table included:

- data relative to the identification of the paper: title, author, journal, year of publication, country
- data relative to the design of the study: type of study, start and end dates, total study duration, name of the cohort and duration of follow-up if applicable, selection and matching criteria for case–control studies
- data relative to the participants: number of participants, number of patients/controls, age, sex (proportion male/female), BMI, ethnicity
- data relative to the method used: for self-report methods, details about duration and recurrence of record; for objective biomarkers, details about measured variables; elements of diet investigated; validation of the tool
- a short summary of the aims and principal results of the study

### Data synthesis

We summarized the characteristics of the studies based on the origin of the participants, the study design and the methods used. We described the methods in terms of number of publications. For the 24-h urine collections and other timed-urine samples, if the value of at least one among sodium, potassium, urea, oxalate, citrate excretions or urinary volume was reported in a publication, we considered that a urinary biomarker was available. For spot urine, we considered pH in addition to the previously mentioned values. For the blood samples, if the value of at least one of the items among glucose, lipid profile, micronutrients (vitamins and minerals), ferritin, albumin, urea or uric acid was given in the publication, we considered that a blood biomarker was available.

We then described the characteristics of the 24-h urine collections in more detail. For this description, we worked in terms of studies and not publications. Thus, if at least one publication related to the same study described a 24-h urine collection, we considered that it was available in the study.

## Results

**Fig. 1:**
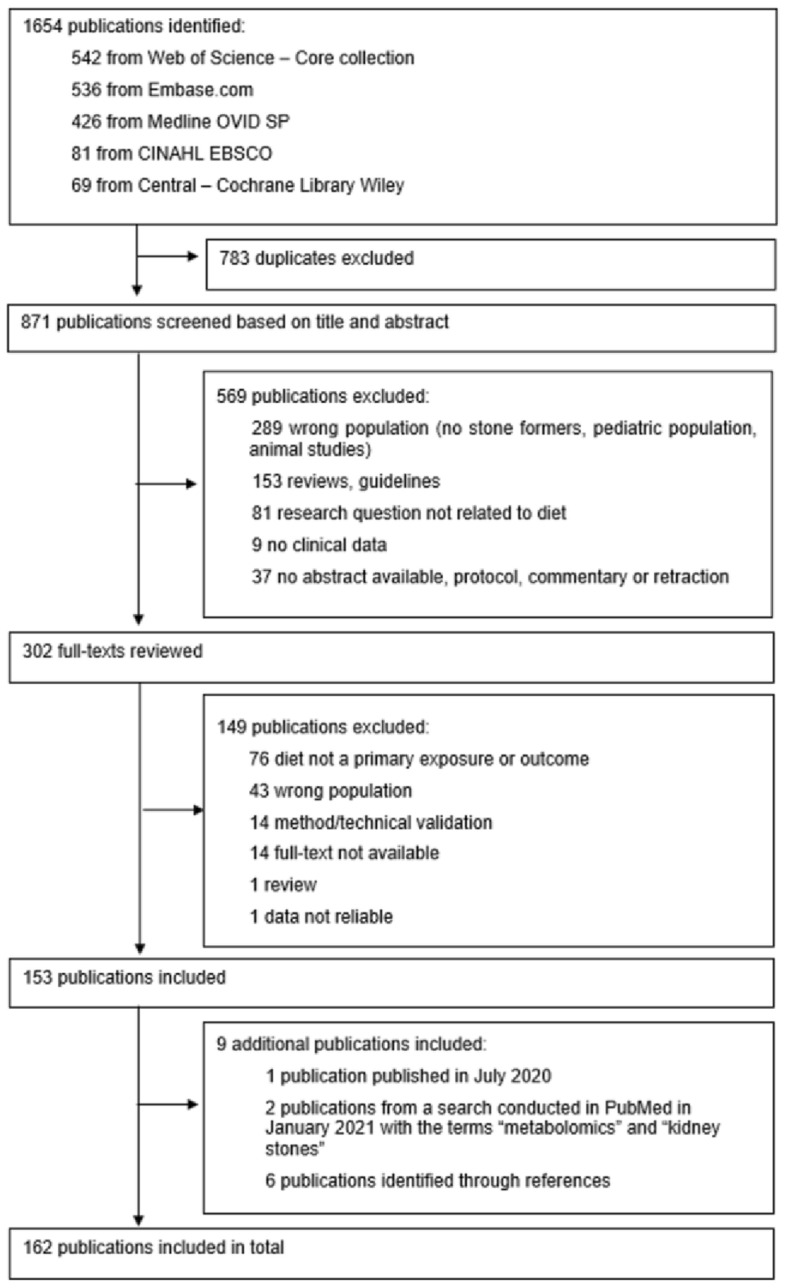
Flow-chart representing the selection process of the publications included in the review

**Fig. 2:**
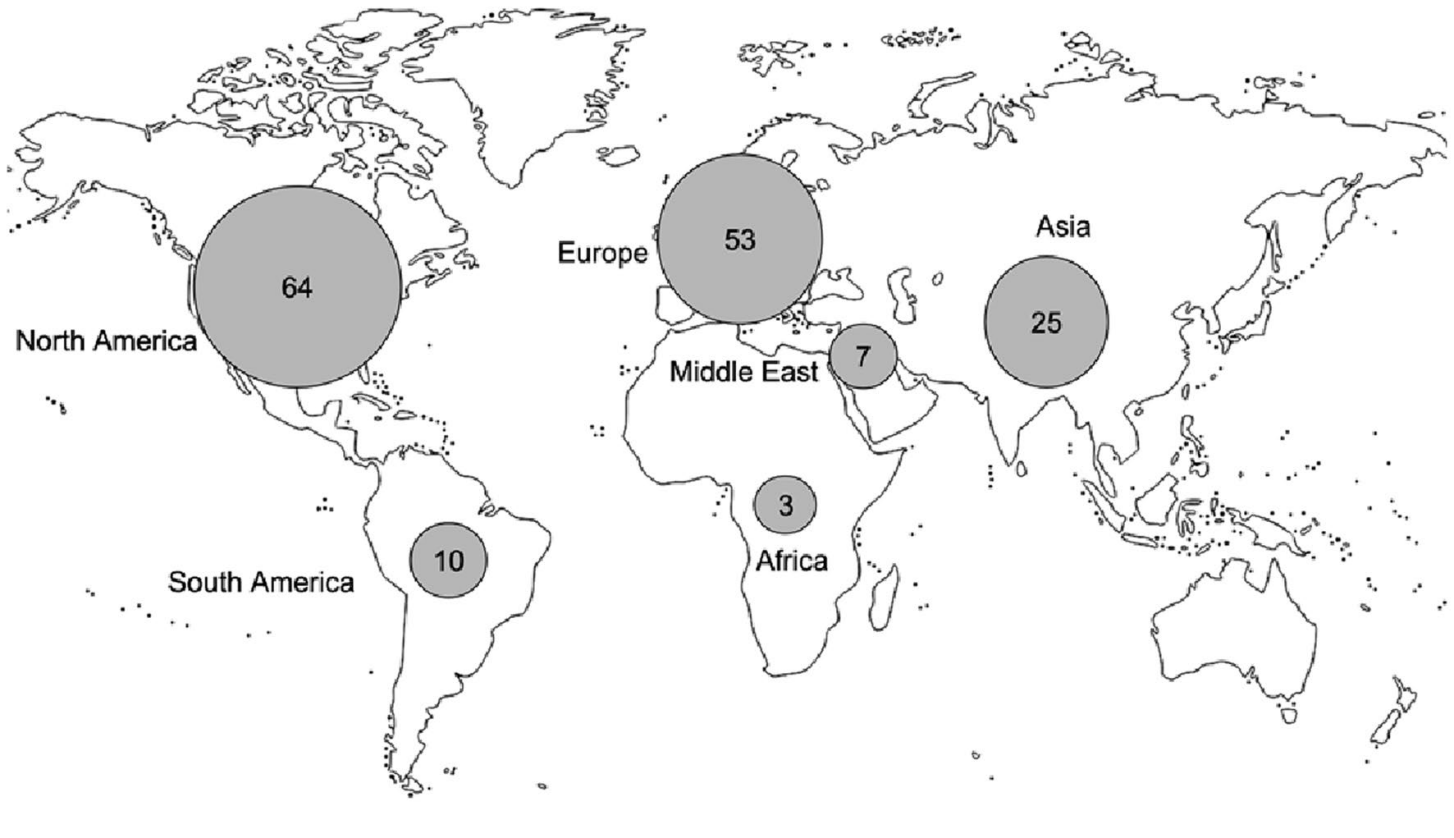
Origin of the participants in the publications. North America region includes Canada, Puerto Rico and the USA. South America region includes Brazil. Europe region includes Austria, Bulgaria, Croatia, Czech Republic, Finland, France, Greece, Germany, Ireland, Italy, Macedonia, Poland, Romania, Serbia, Slovenia, Spain, Sweden and the UK. Middle East region includes Iran, Saudi Arabia and Turkey. Africa region includes Morocco and South Africa. Asia region includes China, India, Japan, Korea, Pakistan, Taiwan and Thailand

We included 162 publications in this review. Several publications were related to the same study, this selection represents 122 independent studies (see Table 1 in the Supplementary material). Figure 1 shows the selection process for the included publications. In most publications, participants were recruited in North America (*n* = 64 publications, 40%) and Europe (*n* = 53 publications, 33%), whereas Asia (*n* = 25 publications, 15%), South America (*n* = 10 publications, 6%), the Middle East (*n* = 7 publications, 4%) and Africa (*n* = 3 publications, 2%) were less represented (Fig. 2).

**Fig. 3:**
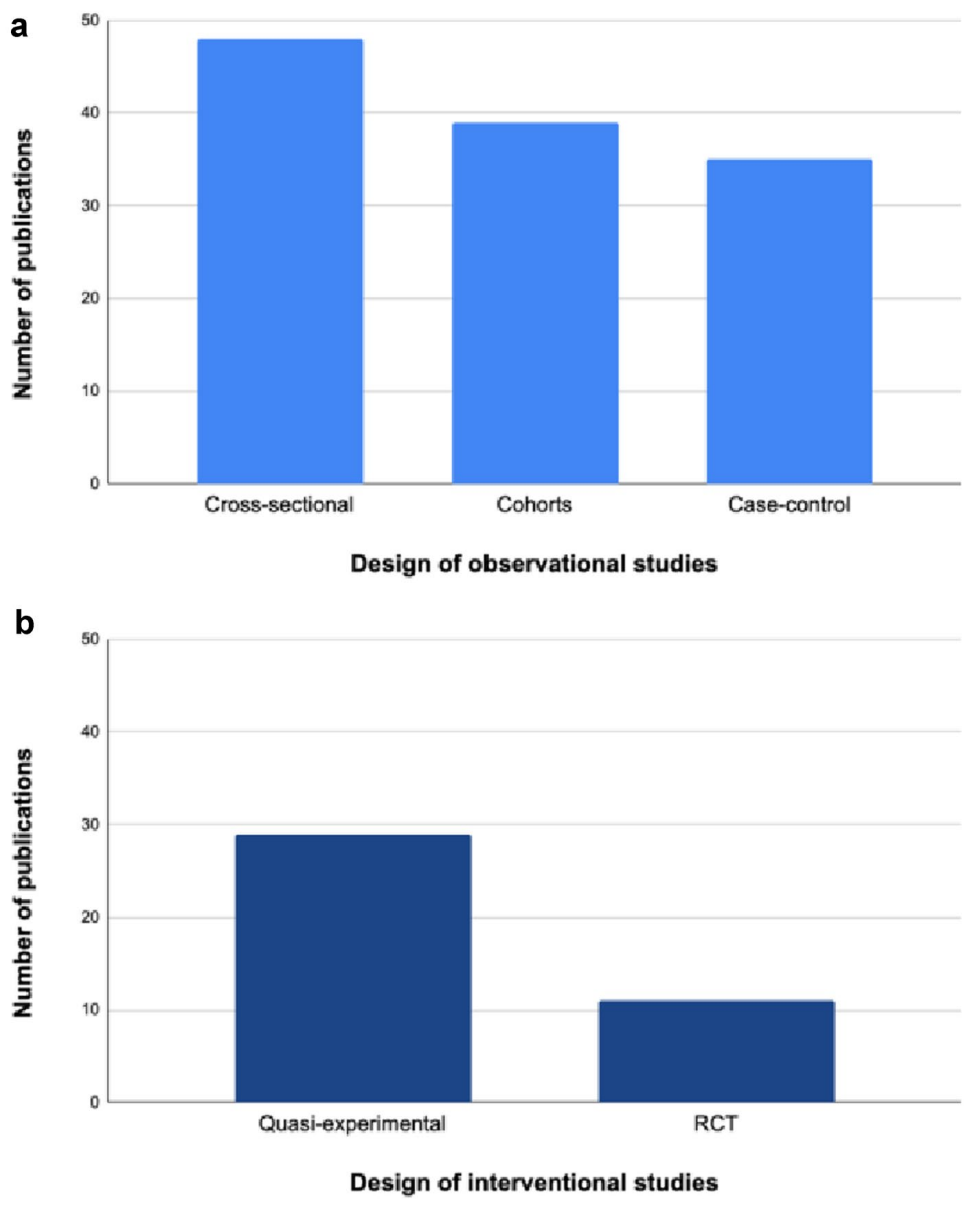
**a** Number of publications for each type of observational studies (*n* = 122); **b** Number of publications for each type of interventional studies (*n* = 40)

The design was observational in 122 publications (75%) and interventional in 40 (25%). Figure 3a shows the number of publications for the different types of observational studies. We split the design of observational studies into cross-sectional studies (*n* = 48 publications, 39%), cohorts (*n* = 39 publications, 32%) and case–control studies (*n* = 35 publications, 29%). Figure 3b represents the number of publications for the different types of interventional studies, split into randomized controlled trials (RCT) (*n* = 11 publications, 27%) and other studies with an experimental setting but without randomization, defined as quasi-experimental (*n* = 29 publications, 73%).

**Fig. 4:**
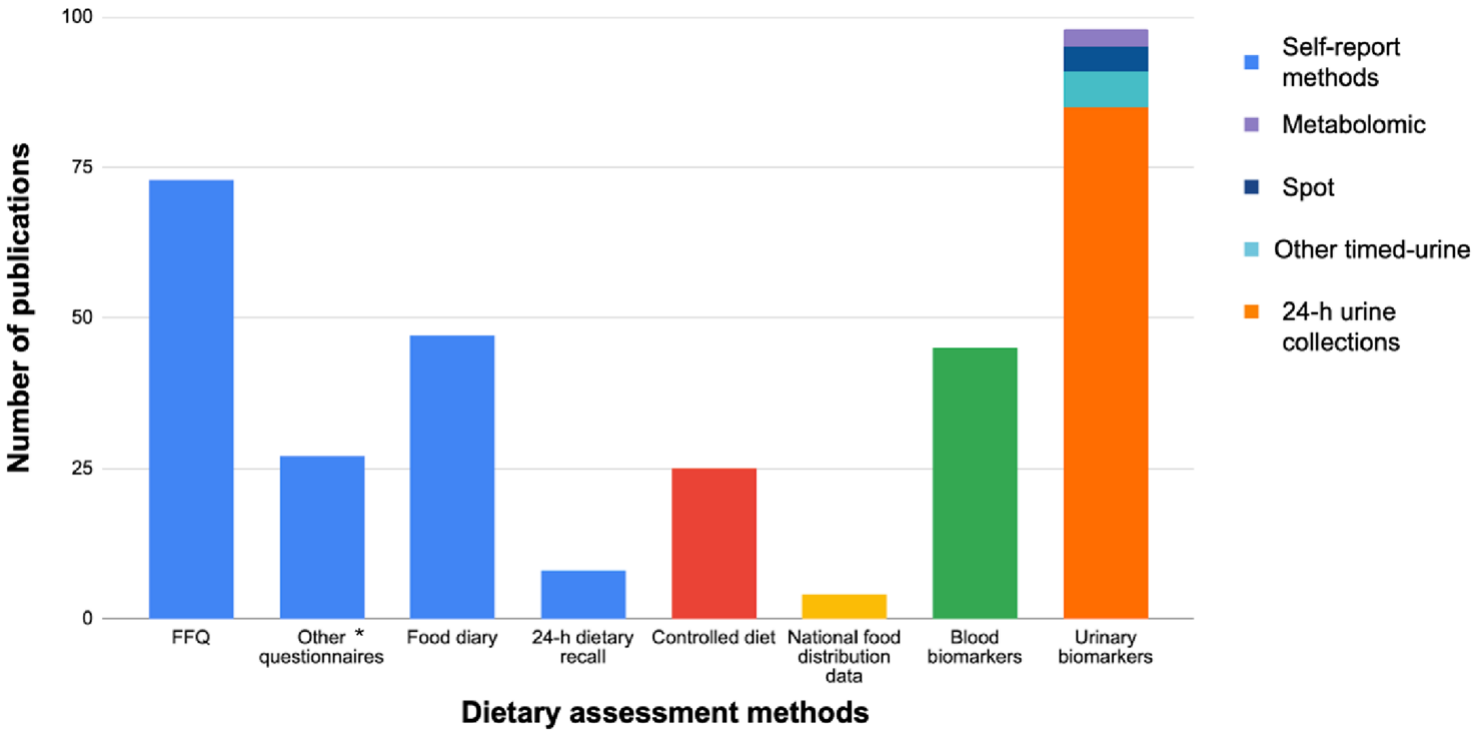
Number of publications per dietary assessment method (*n* = 162).*other questionnaires include diet history and non-FFQ questionnaires

Self-report dietary assessment methods were described in 155 publications (96%) (Fig. 4). In this category, FFQs were the most frequently used (*n* = 73 publications, 47%) and 24-h dietary recalls the least frequently used (*n* = 8 publications, 5%). As shown in Fig. S7 (Supplementary material), 30 publications using a FFQ were related to the Nurses’ Health Study I, II and Health Professionals Follow-Up Studies.

There are different types of FFQs. Some FFQs look only at the frequency of consumption, whereas semi-quantitative FFQs look at the frequency as well as the portions consumed. For FFQs without details about portions, it is still possible to obtain dietary intake by applying standard size portions [26]. However, semi-quantitative FFQs allow for a more precise estimation of the daily intakes. The authors described the FFQs as semi-quantitative in 53 publications (including the 30 publications related to the NHS and HPFS studies that used similar FFQs). In two publications, only the beverages were quantified. In one publication, another self-report method was used to obtain the quantities and was combined with the FFQ to generate the intake, and in 3 publications, the investigators reported the frequency of consumption but the intake. Finally, in the other publications (*n* = 14), we could not determine if the FFQ was semi-quantitative or if a standard size portion had been applied afterwards to generate the intake. This shows the importance of precisely describing the method used. It also calls for a standardization of the description of such method.

Food diaries were used in 47 publications (30%) and other questionnaires in 27 publications (17%). Food diaries were collected for a period of 7 days in 8 publications (17%), 4 days in 5 publications (11%), 3 days in 24 publications (51%) and 1 day in 5 publications (11%). Participants were placed under controlled diets in 25 publications (15%). Only a few studies (*n* = 4 publications, 2%) used regional or national food distribution data or household food purchases registries to study the diet.

The value for at least one urinary biomarker was indicated in 95 publications (59%), with 24-h urine collections for 85 publications (89%), other timed-urine for 6 publications (6%) and spot urine samples for 4 publications (4%). The three metabolomic studies included urine samples. The value for at least one blood biomarker was indicated in 45 publications (28%).

**Fig. 5:**
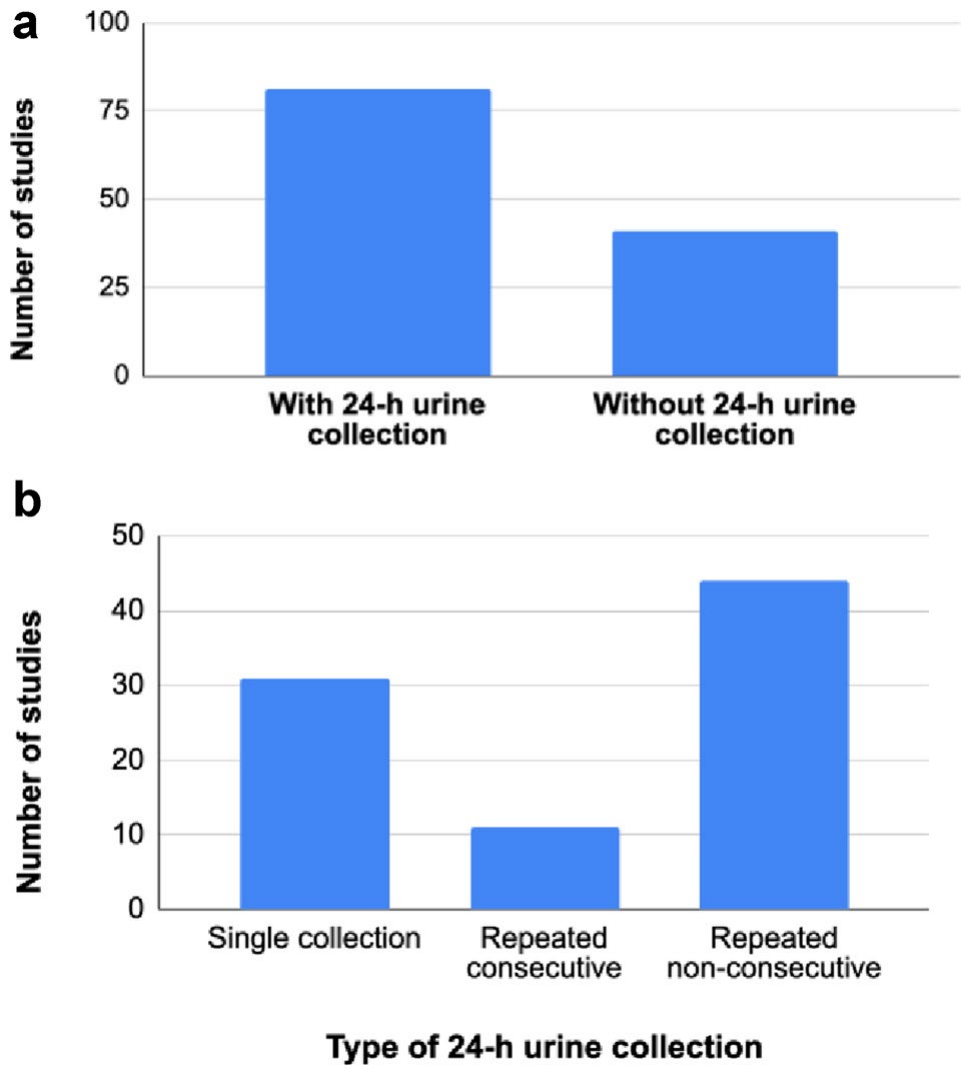
**a** Number of studies with or without 24-h urine collections available (*n* = 122); **b** Number of studies per type of 24-h urine collection (*n* = 81)

In the following sections, we considered the 24-h urine collections in terms of studies and not publications**.** Figure 5a indicates the number of studies with and without 24-h urine collections and Fig. 5b shows the breakdown of the different types of collections performed: single collection and repeated consecutive or non-consecutive collections. Twenty-four hour urine collections were available in 81 studies (66%), while 41 studies (34%) did not have 24-h urine collections.

Most studies with 24-h urine had repeated collections: 11 studies (14%) had repeated consecutive and 44 studies (54%) had repeated non-consecutive collections. All 11 studies with repeated consecutive collections were performed during two consecutive days. In four studies, both repeated consecutive and repeated non-consecutive collections were done. Concerning the non-consecutive repeated collections, the time interval between the collections was not always reported and when reported, it was highly variable and depended on the study design. Finally, 31 studies (38%) had a single 24-h urine collection.

**Fig. 6:**
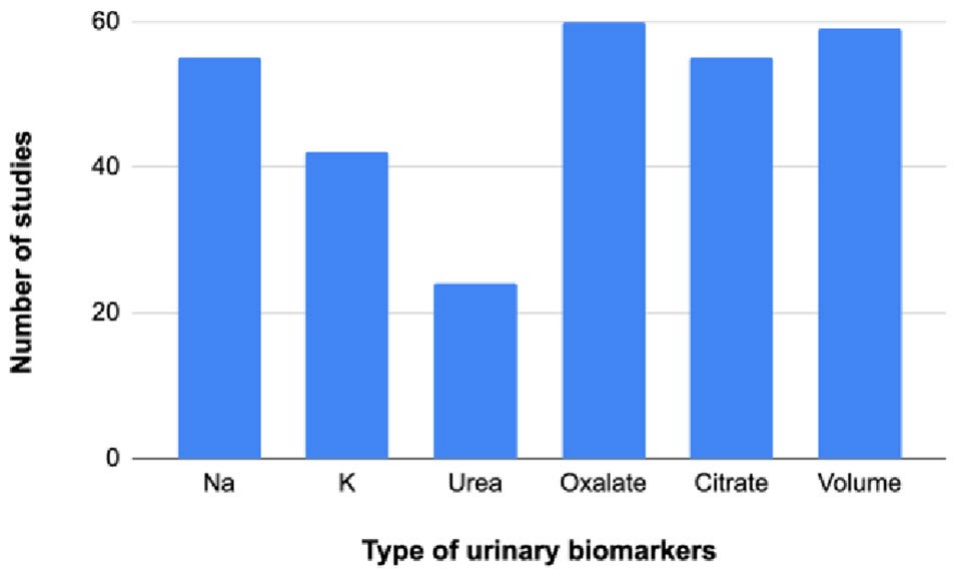
Number of studies per type of urinary biomarkers measured in the 24-h urine collections (*n* = 81). The number of studies represents the studies in which the values of the biomar kers were reported in at least one publication based on this study

Figure 6 shows the number of studies with results on 24-h urinary biomarkers. Excretion rate was reported for sodium (55 studies), potassium (42 studies), urea (24 studies), oxalate (60 studies), citrate (55 studies) and urinary volume (59 studies).

## Discussion

To the best of our knowledge, this is the first scoping review addressing the methods used to evaluate the diet of kidney stone formers. We identified reviews on dietary assessment methods but they were not focused on kidney stone formers [16, 27, 28].

Short and self-addressed dietary assessment methods, such as FFQs or other questionnaires were preferred over methods that required more time or resources, such as 24-h dietary recalls. Previous reviews [16, 27, 28] also showed that FFQs were the most common choice to evaluate dietary intake and that 24-h dietary recalls were less often performed.

FFQs and other questionnaires consist of a pre-established and close-ended set of questions about food and beverage consumption [12] and are developed for a specific research question and a given population [12]. A questionnaire developed for a study can focus on certain aspects of the diet or be more generic, depending on the aim of the study [11]. The validity of FFQs on different populations can thus be limited due to cultural specificities and their validity should be assessed before using them in a new setting [9, 29, 30]. Methods for the validation of FFQs are described in the literature [29, 31].

Of the 73 publications that used FFQs, 54 (74%) specified that the FFQ was validated (30 of which were linked to the same study and used the same FFQ) and 19 publications (26%) did not. Few details on the development and validation process were provided for other questionnaires. Overall, the description of the method used varied across studies. Details on the development of FFQs and other questionnaires, in particular for which population they were developed or their validity, were not available for all studies. This calls for the development of guidelines on how to prepare, validate and report FFQs in future studies.

Food diaries and 24-h dietary recalls seem to be rarely used to evaluate dietary intake in nutritional studies [16, 27] but are often used as references in validation studies [28]. We found that food diaries were used in nearly a third of the studies. Pragmatic aspects arising from 24-h urine collection performed in stone formers might favor this method. Indeed, when collected simultaneously, it is possible to compare nutritional data from the food diaries and urinary and objective biomarkers measured in 24-h urine collections.

We included specific terms in the search equations for new technologies such as “online questionnaire* OR photo app* OR photo phone app* OR smart bottle*”. Several studies mentioned online questionnaires or web applications but overall, even in the more recent papers, new technologies do not seem to be frequently used for the dietary assessment of kidney stone formers. As diet and its links to various health issues are increasingly being studied nowadays, new technologies could help improve dietary assessment [32, 33]. It would be interesting to follow the use of those tools in kidney stone research in future reviews.

Twenty-four hour urine collections are used for the metabolic evaluation of kidney stone formers [8] and are often done in both clinical and research settings. In most studies included in our review, 24-h urine collections were available, but the type of collection varied (single, repeated consecutive or non-consecutive). It is important to check the quality and completeness of the collections before analyzing their composition and measuring objective nutritional biomarkers [34]. Several criteria exist to assess the quality of 24-h urine collections [35–37]. We observed that the criteria used to evaluate the quality and completeness of the 24-h urine collections varied across studies.

We considered 24-h urinary nitrogen, sodium, potassium, volume, oxalate and citrate as objective nutritional biomarkers. 24-h urinary nitrogen (referred to as urea in our review), sodium and potassium are accurate proxies for the dietary intake of protein, sodium and potassium, respectively [38–41]. Urinary oxalate is mainly derived from endogenous metabolism [42, 43] but a previous study showed that dietary consumption could contribute up to 50% of the urinary oxalate excretion [43]. Similarly, diet has an impact on citrate excretion [44] and dietary interventions can be used in case of hypocitraturia [45]. Finally, urinary volume was found to correlate with volume intake [46].

We found that oxalate and citrate excretions were frequently assessed, while urea was rarely reported [38]. Overall, the choice of biomarkers in 24-h urine collections is not standardized and still a matter of debate [47]. New urinary biomarkers have been identified [44] and metabolomic studies are promising. For instance, a study identified a urinary amino acid profile specific to kidney stone formers [25].

Overall, self-report methods, especially FFQs and other questionnaires, are widely used in research. Indeed, FFQs are a time-saving and cost-effective method that can be easily administered to a large number of participants [12]. Yet, as mentioned previously, these types of questionnaires cover only a set of pre-determined foods and beverages and should be validated before use [12]. On the other hand, food diaries or 24-h dietary recalls require more resources but can capture in detail foods and beverages consumed over a short period [9]. However, a single day diary or recall does not provide a good representation of usual dietary intakes [9]. Moreover, all self-report methods are subject to error and biases [9, 48], for instance when measuring protein or total energy intake [48, 49]. Some recommendations have been developed to correct for possible sources of errors when using those methods, for instance combining with objective biomarkers or using statistical methods to generate the usual intake [9, 48, 50]. The 24-h dietary recalls are considered the least biased of this category and the best instrument to measure dietary intake as well as to look at associations between diet and health, but they need to be repeated several times to provide better insight on usual dietary intakes [10].

There are different types of objective nutritional biomarkers [9–12, 14]. Recovery biomarkers, such as 24 h urinary nitrogen, sodium or potassium, are directly related to dietary intake [9–11, 38–40]. However, investigators found that 24-h urine values of sodium and potassium do not reflect individual sodium and potassium intake well, unless repeated collections are performed [41]. Other objective biomarkers such as predictive (e.g. 24-h urinary fructose and sucrose) or concentration (e.g. fatty acids measured in adipose tissues or vitamins in blood) biomarkers are correlated with intake but can be affected by individual metabolism [9, 11]. Objective biomarkers are thus an interesting tool to validate or to measure dietary intake more precisely [9, 11, 14] but, those markers still have limitations and for now, only a limited number are available. Recommendations for future research are to combine several methods, either two self-report methods such as FFQs and 24-h dietary recalls or self-report methods and objective biomarkers [10–12].

The metabolic evaluation of kidney stone formers in clinical practice is complex and includes medical and nutritional history to identify environmental, metabolic and genetic risk factors but also laboratory analyses (24-h urine and serum, stone composition) [8, 51–53]. Guidelines have been published regarding indications for metabolic evaluation and recurrence prevention [53] depending on the population (high-risk or low-risk stone formers) and the type of stone.

Many studies were conducted in North America or in Europe and knowledge in this domain mostly comes from large American cohorts [54–56]. However, diet is highly variable across populations [9, 30, 57, 58] and it would be important to verify whether the same dietary recommendations are valid in other countries.

Furthermore, most studies had an observational design and among interventional studies, there were few RCTs. Interventional nutritional studies are more difficult to conduct as blinding and randomization are not always feasible. It is difficult to plan and maintain RCTs over long periods. RCTs also usually do not reflect real-life settings and have therefore limited external validity.

Finally, many studies in our review relied on punctual dietary assessment, with cross-sectional studies or single 24-h urine collections and did not evaluate diet longitudinally. This is a clear limitation for usual food intake evaluation. Indeed, long-term diet is an important exposure for surveillance and epidemiology to study health-related outcomes [9].

We included various study designs to have an overview of the literature and considered many research questions and approaches. With the different methodologies in our selection, certain methods may be appropriate for a given purpose but not for another. Hence, we cannot draw a general conclusion concerning the different methods that would be applicable to all study designs. Moreover, we conducted a systematic search of the literature but it is possible that we missed some publications of interest.

## Conclusion

Given the role of diet in kidney stone formation, it is important to know how research is conducted in this field to inform future studies. Self-report methods and especially FFQs are the most frequently used and knowledge in this field is mainly based on observational data and Western diets. Overall, we observed that there is heterogeneity in the methodology description.

We thus want to stress the importance of precisely reporting the methodology used to collect dietary data, as it is a key element to interpret the results and build evidence. In addition, it is important to evaluate the impact of different diets on stone formation and when possible to try to implement longitudinal or interventional studies. Finally, the combination of self-report methods with objective dietary biomarkers, including blood and urine metabolomic analyses, as well as smartphone applications to take pictures of meals will represent the best way forward.

## Supplementary Information

Below is the link to the electronic supplementary material.

Supplementary file1 (DOCX 20 kb)

Supplementary file2 (DOCX 181 kb)

Supplementary file3 (PDF 113 kb)
