# Supplementary material for: Methods for the dietary assessment of adult kidney stone formers: a scoping review
Source: J Nephrol. 2022 Feb 15;35(3):821–30. doi: 10.1007/s40620-022-01259-3 (PMC8995246; doi:10.1007/s40620-022-01259-3)
Supplement: Supplementary file 1 — Supplementary file1 (DOCX 20 kb) [file 40620_2022_1259_MOESM1_ESM.docx]

**Methods for the Dietary Assessment of Adult Kidney Stone Formers: A Scoping Review**

**Journal of Nephrology**

Constance Legay^1,3,4^, Tropoja Krasniqi^1,2^, Alice Bourdet^3^, Olivier Bonny^1,2,4^ and Murielle Bochud^3,4^

^1^ Department of Biomedical Sciences, University of Lausanne, Lausanne, Switzerland

^2^ Service of Nephrology, Lausanne University Hospital, Lausanne, Switzerland

^3^ Department of Epidemiology and Health Systems, Unisanté, Lausanne, Switzerland

^4^ NCCR Kidney.CH

Corresponding author: [Olivier.Bonny@unil.ch](mailto:Olivier.Bonny@unil.ch)

## Full search equations

**Medline Ovid SP**

(Urolithiasis/ OR exp Nephrolithiasis/ OR kidney lithiasis.ti,ab,kf. OR nephrolithiasis.ti,ab,kf. OR renal lithiasis.ti,ab,kf. OR renolithiasis.ti,ab,kf. OR ((kidney OR renal) adj2 (calcul* OR stone*)).ti,ab,kf. OR (urinary calcul* OR urinary lithiasis OR urinary stone* OR urinary tract calcul* OR urinary tract lithiasis OR urinary tract stone* OR urine calcul* OR urine lithiasis OR urine stone* OR uro-lithiasis OR urocalcul* OR urolith OR urolithiasis OR urolithogenesis OR urologic calcul* OR urological calcul*).ti,ab,kf.) AND (Nutrition Assessment/ OR Diet Records/ OR Mobile Applications/ OR ((diet* OR eating OR fluid consumption OR fluid intake OR food OR nutrient* OR nutrition*) adj3 (assess* OR behavio$r* OR biochemical analysis OR biochemistry OR diaries OR diary OR evaluat* OR habit$ OR measur* OR record*)).ti,ab,kf. OR ((biological marker* OR biomarker*).ti,ab,kf,sh. AND (diet* OR food OR nutrit*).ti,ab,kf,hw.) OR (24h recall OR 24hour recall OR 24-hour recall OR 24h urine collection OR 24hour urine collection OR 24-hour urine collection OR FFQ OR food frequency questionnaire OR online questionnaire* OR photo app* OR photo phone app* OR smart bottle*).ti,ab,kf.) AND English.lg. NOT (exp animals/ not humans/) NOT ((exp Infant/ OR exp Child/ OR Adolescent/) not exp Adult/) NOT (comment/ or editorial/ or letter/)

As of June 10th 2020, 426 references found.

**Embase.com**

('urolithiasis'/de OR 'nephrolithiasis'/de OR 'kidney lithiasis':ti,ab,kw OR 'nephrolithiasis':ti,ab,kw OR 'renal lithiasis':ti,ab,kw OR 'renolithiasis':ti,ab,kw OR ((kidney OR renal) NEAR/2 (calcul* OR stone*)):ti,ab,kw OR ('urinary calcul*' OR 'urinary lithiasis' OR 'urinary stone*' OR 'urinary tract calcul*' OR 'urinary tract lithiasis' OR 'urinary tract stone*' OR 'urine calcul*' OR 'urine lithiasis' OR 'urine stone*' OR 'uro-lithiasis' OR urocalcul* OR urolith OR urolithiasis OR urolithogenesis OR 'urologic calcul*' OR 'urological calcul*'):ti,ab,kw) AND ('nutritional assessment'/de OR 'food frequency questionnaire'/de OR 'mobile application'/exp OR ((diet* OR eating OR 'fluid consumption' OR 'fluid intake' OR food OR nutrient* OR nutrition*) NEAR/3 (assess* OR behavio$r* OR 'biochemical analysis' OR biochemistry OR diaries OR diary OR evaluat* OR habit$ OR measur* OR record*)):ti,ab,kw OR (('biological marker*' OR biomarker*):ti,ab,kw,de AND (diet* OR food OR nutrit*):ti,ab,kw,de) OR ('24h recall' OR '24hour recall' OR '24-hour recall' OR '24h urine collection' OR '24hour urine collection' OR '24-hour urine collection' OR FFQ OR 'food frequency questionnaire' OR 'online questionnaire*' OR 'photo app*' OR 'photo phone app*' OR 'smart bottle*'):ti,ab,kw) AND [english]/lim NOT ([animals]/lim NOT [humans]/lim) NOT ('juvenile'/exp NOT 'adult'/exp) NOT ('conference abstract'/it OR 'conference review'/it OR 'editorial'/it OR 'letter'/it)

As of June 10th 2020, 536 references found.

**CINAHL EBSCO**

(MH "Urolithiasis" OR TI "kidney lithiasis" OR AB "kidney lithiasis" OR TI nephrolithiasis OR AB nephrolithiasis OR TI "renal lithiasis" OR AB "renal lithiasis" OR TI renolithiasis OR AB renolithiasis OR ((TI kidney OR AB kidney OR TI renal OR AB renal) N2 (TI calcul* OR AB calcul* OR TI stone* OR AB stone*)) OR TI "urinary calcul*" OR AB "urinary calcul*" OR TI "urinary lithiasis" OR AB "urinary lithiasis" OR TI "urinary stone*" OR AB "urinary stone*" OR TI "urinary tract calcul*" OR AB "urinary tract calcul*" OR TI "urinary tract lithiasis" OR AB "urinary tract lithiasis" OR TI "urinary tract stone*" OR AB "urinary tract stone*" OR TI "urine calcul*" OR AB "urine calcul*" OR TI "urine lithiasis" OR AB "urine lithiasis" OR TI "urine stone*" OR AB "urine stone*" OR TI uro-lithiasis OR AB uro-lithiasis OR TI urocalcul* OR AB urocalcul* OR TI urolith OR AB urolith OR TI urolithiasis OR AB urolithiasis OR TI urolithogenesis OR AB urolithogenesis OR TI "urologic calcul*" OR AB "urologic calcul*" OR TI "urological calcul*" OR AB "urological calcul*")

AND

(MH "Nutritional Assessment" OR MH "Diet Records" OR MH "Mobile Applications" OR ((TI diet* OR AB diet* OR TI eating OR AB eating OR TI "fluid consumption" OR AB "fluid consumption" OR TI "fluid intake" OR AB "fluid intake" OR TI food OR AB food OR TI nutrient* OR AB nutrient* OR TI nutrition* OR AB nutrition*) N3 (TI assess* OR AB assess* OR TI behavio#r# OR AB behavior#r# OR TI "biochemical analysis" OR AB "biochemical analysis" OR TI biochemistry OR AB biochemistry OR TI diaries OR AB diaries OR TI diary OR AB diary OR TI evaluat* OR AB evaluat* OR TI habit* OR AB habit* OR TI measur* OR AB measur* OR TI record* OR AB record*)) OR ((TI "biological marker*" OR AB "biological marker*" OR TI biomarker* OR AB biomarker*) AND (TI diet* OR AB diet* OR TI food OR AB food OR TI nutrit* OR AB nutrit*)) OR (TI "24h recall" OR AB "24h recall" OR TI "24hour recall" OR AB "24hour recall" OR TI "24-hour recall" OR AB "24-hour recall" OR TI "24h urine collection" OR AB "24h urine collection" OR TI "24hour urine collection" OR AB "24hour urine collection" OR TI "24-hour urine collection" OR AB "24-hour urine collection" OR TI FFQ OR AB FFQ OR TI "food frequency questionnaire" OR AB "food frequency questionnaire" OR TI "online questionnaire*" OR AB "online questionnaire*" OR TI "photo app*" OR AB "photo app*" OR TI "photo phone app*" OR AB "photo phone app*" OR TI "smart bottle*" OR AB "smart bottle*"))

NOT (((MH "Child+") OR (MH "Adolescence+")) NOT (MH "Adult+"))

As of June 10th 2020, 81 references found.

**Central - Cochrane Library Wiley**

(urolithiasis OR "kidney lithiasis" OR nephrolithiasis OR "renal lithiasis" OR renolithiasis OR ((kidney OR renal) NEAR/2 (calcul* OR stone*)) OR "urinary calcul*" OR "urinary lithiasis" OR "urinary stone*" OR "urinary tract calcul*" OR "urinary tract lithiasis" OR "urinary tract stone*" OR "urine calcul*" OR "urine lithiasis" OR "urine stone*" OR uro-lithiasis OR urocalcul* OR urolith OR urolithiasis OR urolithogenesis OR "urologic calcul*" OR "urological calcul*") AND (((diet* OR eating OR "fluid consumption" OR "fluid intake" OR food OR nutrient* OR nutrition*) NEAR/3 (assess* OR behavior* OR behaviour* OR "biochemical analysis" OR biochemistry OR diaries OR diary OR evaluat* OR habit OR habits OR measur* OR record*)) OR (("biological marker*" OR biomarker*) AND (diet* OR food OR nutrit*)) OR ("24h recall" OR "24hour recall" OR "24-hour recall" OR "24h urine collection" OR "24hour urine collection" OR "24-hour urine collection" OR FFQ OR "food frequency questionnaire" OR "mobile application*" OR "online questionnaire*" OR "photo app*" OR "photo phone app*" OR "smart bottle*"))

As of June 10th 2020, 69 references found.

**Web of Science – Core collection***

(urolithiasis OR "kidney lithiasis" OR nephrolithiasis OR "renal lithiasis" OR renolithiasis OR ((kidney OR renal) NEAR/2 (calcul* OR stone*)) OR "urinary calcul*" OR "urinary lithiasis" OR "urinary stone*" OR "urinary tract calcul*" OR "urinary tract lithiasis" OR "urinary tract stone*" OR "urine calcul*" OR "urine lithiasis" OR "urine stone*" OR uro-lithiasis OR urocalcul* OR urolith OR urolithiasis OR urolithogenesis OR "urologic calcul*" OR "urological calcul*") AND (((diet* OR eating OR "fluid consumption" OR "fluid intake" OR food OR nutrient* OR nutrition*) NEAR/3 (assess* OR behavior* OR behaviour* OR "biochemical analysis" OR biochemistry OR diaries OR diary OR evaluat* OR habit OR habits OR measur* OR record*)) OR (("biological marker*" OR biomarker*) AND (diet* OR food OR nutrit*)) OR ("24h recall" OR "24hour recall" OR "24-hour recall" OR "24h urine collection" OR "24hour urine collection" OR "24-hour urine collection" OR FFQ OR "food frequency questionnaire" OR "mobile application*" OR "online questionnaire*" OR "photo app*" OR "photo phone app*" OR "smart bottle*")) NOT ((Child* OR Adolescen*) NOT adult))

As of June 10th 2020, 542 references found.
