# Supplementary material for: Methods for the dietary assessment of adult kidney stone formers: a scoping review
Source: J Nephrol. 2022 Feb 15;35(3):821–30. doi: 10.1007/s40620-022-01259-3 (PMC8995246; doi:10.1007/s40620-022-01259-3)
Supplement: Supplementary file 2 — Supplementary file2 (DOCX 181 kb) [file 40620_2022_1259_MOESM2_ESM.docx]

**Methods for the Dietary Assessment of Adult Kidney Stone Formers: A Scoping Review**

**Journal of Nephrology**

Constance Legay^1,3,4^, Tropoja Krasniqi^1,2^, Alice Bourdet^3^, Olivier Bonny^1,2,4^ and Murielle Bochud^3,4^

^1^ Department of Biomedical Sciences, University of Lausanne, Lausanne, Switzerland

^2^ Service of Nephrology, Lausanne University Hospital, Lausanne, Switzerland

^3^ Department of Epidemiology and Health Systems, Unisanté, Lausanne, Switzerland

^4^ NCCR Kidney.CH

Corresponding author: [Olivier.Bonny@unil.ch](mailto:Olivier.Bonny@unil.ch)

**Table 1** Description of included studies

| Study Name | Number of publications | Observational design | Interventional design | References |
| --- | --- | --- | --- | --- |
| Studies with multiple publications | 48 | 47 | 1 |  |
| NHS I and II, HPFS ^*^ | 30 | 30 | 0 | [1-30] |
| GENOA ^†^ | 5 | 5 | 0 | [31-35] |
| WHI ^‡^ | 3 | 3 | 0 | [36-38] |
| SUN ^§^ | 2 | 2 | 0 | [39, 40] |
| SWHS and SMHS ^¶^ | 2 | 2 | 0 | [41, 42] |
| Bonn Urolithiasis Follow-up Study | 2 | 1 | 1 | [43, 44] |
| Naya et al. | 2 | 2 | 0 | [45, 46] |
| Damasio et al. | 2 | 2 | 0 | [47, 48] |
| Studies with a single publication | 114 | 75 | 39 | [49-162] |
| Total | 162 | 122 | 40 |  |

^*^ Nurses’ Health Study I and II, Health Professionals Follow-Up Study

^†^ The Genetic Epidemiology Network of Arteriopathy cohort

^‡^ The Women's Health Initiative Observational Study

^§^ The Seguimiento Universidad de Navarra

^¶^ Shanghai Women's Health Study and Shanghai Men's Health Study

**References**

1. Curhan GC, Rimm EB, Willett WC, et al (1994) Regional variation in nephrolithiasis incidence and prevalence among United States men. The Journal of urology 151:838-841 <https://doi.org/10.1016/s0022-5347(17)35101-7>

2. Curhan GC, Willett WC, Knight EL, et al (2004) Dietary factors and the risk of incident kidney stones in younger women: Nurses' Health Study II. Archives of internal medicine 164:885-891 <https://doi.org/10.1001/archinte.164.8.885>

3. Curhan GC, Willett WC, Rimm EB, et al (1998) Body size and risk of kidney stones. Journal of the American Society of Nephrology : JASN 9:1645-1652

4. Curhan GC, Willett WC, Rimm EB, et al (1996) Prospective study of beverage use and the risk of kidney stones. American journal of epidemiology 143:240-247 <https://doi.org/10.1093/oxfordjournals.aje.a008734>

5. Curhan GC, Willett WC, Rimm EB, et al (1996) A prospective study of the intake of vitamins C and B6, and the risk of kidney stones in men. The Journal of urology 155:1847-1851

6. Curhan GC, Willett WC, Rimm EB, et al (1993) A prospective study of dietary calcium and other nutrients and the risk of symptomatic kidney stones. The New England journal of medicine 328:833-838 <https://doi.org/10.1056/nejm199303253281203>

7. Curhan GC, Willett WC, Rimm EB, et al (1997) Family history and risk of kidney stones. Journal of the American Society of Nephrology : JASN 8:1568-1573

8. Curhan GC, Willett WC, Speizer FE, et al (1997) Comparison of dietary calcium with supplemental calcium and other nutrients as factors affecting the risk for kidney stones in women. Annals of internal medicine 126:497-504 <https://doi.org/10.7326/0003-4819-126-7-199704010-00001>

9. Curhan GC, Willett WC, Speizer FE, et al (1998) Beverage use and risk for kidney stones in women. Annals of internal medicine 128:534-540 <https://doi.org/10.7326/0003-4819-128-7-199804010-00003>

10. Ferraro PM, Curhan GC, Gambaro G, et al (2016) Total, Dietary, and Supplemental Vitamin C Intake and Risk of Incident Kidney Stones. American journal of kidney diseases : the official journal of the National Kidney Foundation 67:400-407 <https://doi.org/10.1053/j.ajkd.2015.09.005>

11. Ferraro PM, Curhan GC, Sorensen MD, et al (2015) Physical activity, energy intake and the risk of incident kidney stones. The Journal of urology 193:864-868 <https://doi.org/10.1016/j.juro.2014.09.010>

12. Ferraro PM, Gambaro G, Curhan GC, et al (2018) Intake of Trace Metals and the Risk of Incident Kidney Stones. The Journal of urology 199:1534-1539 <https://doi.org/10.1016/j.juro.2018.01.077>

13. Ferraro PM, Mandel EI, Curhan GC, et al (2016) Dietary Protein and Potassium, Diet-Dependent Net Acid Load, and Risk of Incident Kidney Stones. Clinical journal of the American Society of Nephrology : CJASN 11:1834-1844 <https://doi.org/10.2215/cjn.01520216>

14. Ferraro PM, Taylor EN, Gambaro G, et al (2018) Vitamin B6 intake and the risk of incident kidney stones. Urolithiasis 46:265-270 <https://doi.org/10.1007/s00240-017-0999-5>

15. Ferraro PM, Taylor EN, Gambaro G, et al (2013) Soda and other beverages and the risk of kidney stones. Clinical journal of the American Society of Nephrology : CJASN 8:1389-1395 <https://doi.org/10.2215/cjn.11661112>

16. Ferraro PM, Taylor EN, Gambaro G, et al (2014) Caffeine intake and the risk of kidney stones. The American journal of clinical nutrition 100:1596-1603 <https://doi.org/10.3945/ajcn.114.089987>

17. Ferraro PM, Taylor EN, Gambaro G, et al (2017) Dietary and Lifestyle Risk Factors Associated with Incident Kidney Stones in Men and Women. The Journal of urology 198:858-863 <https://doi.org/10.1016/j.juro.2017.03.124>

18. Ferraro PM, Taylor EN, Gambaro G, et al (2017) Vitamin D Intake and the Risk of Incident Kidney Stones. The Journal of urology 197:405-410 <https://doi.org/10.1016/j.juro.2016.08.084>

19. Mandel EI, Taylor EN, Curhan GC (2013) Dietary and lifestyle factors and medical conditions associated with urinary citrate excretion. Clinical journal of the American Society of Nephrology : CJASN 8:901-908 <https://doi.org/10.2215/cjn.07190712>

20. Rodriguez A, Curhan GC, Gambaro G, et al (2020) Mediterranean diet adherence and risk of incident kidney stones. The American journal of clinical nutrition <https://doi.org/10.1093/ajcn/nqaa066>

21. Taylor EN, Curhan GC (2013) Dietary calcium from dairy and nondairy sources, and risk of symptomatic kidney stones. The Journal of urology 190:1255-1259 <https://doi.org/10.1016/j.juro.2013.03.074>

22. Taylor EN, Curhan GC (2008) Determinants of 24-hour urinary oxalate excretion. Clinical journal of the American Society of Nephrology : CJASN 3:1453-1460 <https://doi.org/10.2215/cjn.01410308>

23. Taylor EN, Curhan GC (2007) Oxalate intake and the risk for nephrolithiasis. Journal of the American Society of Nephrology : JASN 18:2198-2204 <https://doi.org/10.1681/asn.2007020219>

24. Taylor EN, Curhan GC (2008) Fructose consumption and the risk of kidney stones. Kidney international 73:207-212 <https://doi.org/10.1038/sj.ki.5002588>

25. Taylor EN, Fung TT, Curhan GC (2009) DASH-style diet associates with reduced risk for kidney stones. Journal of the American Society of Nephrology 20:2253-2259

26. Taylor EN, Stampfer MJ, Curhan GC (2004) Dietary factors and the risk of incident kidney stones in men: new insights after 14 years of follow-up. Journal of the American Society of Nephrology : JASN 15:3225-3232 <https://doi.org/10.1097/01.Asn.0000146012.44570.20>

27. Taylor EN, Stampfer MJ, Curhan GC (2005) Fatty acid intake and incident nephrolithiasis. American journal of kidney diseases : the official journal of the National Kidney Foundation 45:267-274 <https://doi.org/10.1053/j.ajkd.2004.09.026>

28. Taylor EN, Stampfer MJ, Curhan GC (2005) Obesity, weight gain, and the risk of kidney stones. Jama 293:455-462 <https://doi.org/10.1001/jama.293.4.455>

29. Taylor EN, Stampfer MJ, Curhan GC (2005) Diabetes mellitus and the risk of nephrolithiasis. Kidney international 68:1230-1235 <https://doi.org/10.1111/j.1523-1755.2005.00516.x>

30. Taylor EN, Stampfer MJ, Mount DB, et al (2010) DASH-style diet and 24-hour urine composition. Clinical journal of the American Society of Nephrology : CJASN 5:2315-2322 <https://doi.org/10.2215/cjn.04420510>

31. Lieske JC, Turner ST, Edeh SN, et al (2014) Heritability of urinary traits that contribute to nephrolithiasis. Clinical journal of the American Society of Nephrology : CJASN 9:943-950 <https://doi.org/10.2215/cjn.08210813>

32. Lieske JC, Turner ST, Edeh SN, et al (2016) Heritability of dietary traits that contribute to nephrolithiasis in a cohort of adult sibships. Journal of nephrology 29:45-51 <https://doi.org/10.1007/s40620-015-0204-2>

33. Perinpam M, Ware EB, Smith JA, et al (2016) Key influence of sex on urine volume and osmolality. Biology of sex differences 7:12 <https://doi.org/10.1186/s13293-016-0063-0>

34. Perinpam M, Ware EB, Smith JA, et al (2015) Effect of Demographics on Excretion of Key Urinary Factors Related to Kidney Stone Risk. Urology 86:690-696 <https://doi.org/10.1016/j.urology.2015.07.012>

35. Perinpam M, Ware EB, Smith JA, et al (2017) Association of urinary citrate excretion, pH, and net gastrointestinal alkali absorption with diet, diuretic use, and blood glucose concentration. Physiological reports 5<https://doi.org/10.14814/phy2.13411>

36. Sorensen MD, Chi T, Shara NM, et al (2014) Activity, energy intake, obesity, and the risk of incident kidney stones in postmenopausal women: a report from the Women's Health Initiative. Journal of the American Society of Nephrology : JASN 25:362-369 <https://doi.org/10.1681/asn.2013050548>

37. Sorensen MD, Hsi RS, Chi T, et al (2014) Dietary intake of fiber, fruit and vegetables decreases the risk of incident kidney stones in women: a Women's Health Initiative report. The Journal of urology 192:1694-1699 <https://doi.org/10.1016/j.juro.2014.05.086>

38. Sorensen MD, Kahn AJ, Reiner AP, et al (2012) Impact of nutritional factors on incident kidney stone formation: a report from the WHI OS. The Journal of urology 187:1645-1649 <https://doi.org/10.1016/j.juro.2011.12.077>

39. Carlos S, De La Fuente-Arrillaga C, Bes-Rastrollo M, et al (2018) Mediterranean Diet and Health Outcomes in the SUN Cohort. Nutrients 10<https://doi.org/10.3390/nu10040439>

40. Leone A, Fernández-Montero A, de la Fuente-Arrillaga C, et al (2017) Adherence to the Mediterranean Dietary Pattern and Incidence of Nephrolithiasis in the Seguimiento Universidad de Navarra Follow-up (SUN) Cohort. American journal of kidney diseases : the official journal of the National Kidney Foundation 70:778-786 <https://doi.org/10.1053/j.ajkd.2017.06.027>

41. Shu X, Cai H, Xiang YB, et al (2019) Green tea intake and risk of incident kidney stones: Prospective cohort studies in middle-aged and elderly Chinese individuals. International journal of urology : official journal of the Japanese Urological Association 26:241-246 <https://doi.org/10.1111/iju.13849>

42. Shu X, Calvert JK, Cai H, et al (2019) Plant and Animal Protein Intake and Risk of Incident Kidney Stones: Results from the Shanghai Men's and Women's Health Studies. The Journal of urology 202:1217-1223 <https://doi.org/10.1097/ju.0000000000000493>

43. Siener R, Ebert D, Nicolay C, et al (2003) Dietary risk factors for hyperoxaluria in calcium oxalate stone formers. Kidney international 63:1037-1043 <https://doi.org/10.1046/j.1523-1755.2003.00807.x>

44. Siener R, Glatz S, Nicolay C, et al (2003) Prospective study on the efficacy of a selective treatment and risk factors for relapse in recurrent calcium oxalate stone patients. European urology 44:467-474 <https://doi.org/10.1016/s0302-2838(03)00317-8>

45. Naya Y, Ito H, Masai M, et al (2000) Effect of dietary intake on urinary oxalate excretion in calcium oxalate stone formers in their forties. European urology 37:140-144 <https://doi.org/10.1159/000020130>

46. Naya Y, Ito H, Masai M, et al (2002) Association of dietary fatty acids with urinary oxalate excretion in calcium oxalate stone-formers in their fourth decade. BJU international 89:842-846 <https://doi.org/10.1046/j.1464-410x.2002.02740.x>

47. Damasio PC, Amaro CR, Berto SJ, et al (2010) Urinary lithiasis and idiopathic hypercalciuria: the importance of dietary intake evaluation. International braz j urol : official journal of the Brazilian Society of Urology 36:557-562 <https://doi.org/10.1590/s1677-55382010000500005>

48. Damasio PC, Amaro CR, Cunha NB, et al (2011) The role of salt abuse on risk for hypercalciuria. Nutrition journal 10:3 <https://doi.org/10.1186/1475-2891-10-3>

49. Al Zahrani H, Norman RW, Thompson C, et al (2000) The dietary habits of idiopathic calcium stone-formers and normal control subjects. BJU international 85:616-620 <https://doi.org/10.1046/j.1464-410x.2000.00511.x>

50. Allen SE, Singh S, Robertson WG (2006) The increased risk of urinary stone disease in betel quid chewers. Urological research 34:239-243 <https://doi.org/10.1007/s00240-006-0050-8>

51. Baatiah NY, Alhazmi RB, Albathi FA, et al (2020) Urolithiasis: Prevalence, risk factors, and public awareness regarding dietary and lifestyle habits in Jeddah, Saudi Arabia in 2017. Urology annals 12:57-62 <https://doi.org/10.4103/ua.Ua_13_19>

52. Bailly GG, Norman RW, Thompson C (2000) Effects of dietary fat on the urinary risk factors of calcium stone disease. Urology 56:40-44 <https://doi.org/10.1016/s0090-4295(00)00590-2>

53. Barker DJP, Morris JA, Margetts BM (1988) Diet and renal stones in 72 areas in England and Wales. British journal of urology 62:315-318

54. Basiri A, Shakhssalim N, Khoshdel AR, et al (2009) Influential nutrient in urolithiasis incidence: protein or meat? Journal of renal nutrition : the official journal of the Council on Renal Nutrition of the National Kidney Foundation 19:396-400 <https://doi.org/10.1053/j.jrn.2009.01.017>

55. Bazyar H, Ahmadi A, Zare Javid A, et al (2019) The association between dietary intakes and stone formation in patients with urinary stones in Shiraz. Medical journal of the Islamic Republic of Iran 33:8 <https://doi.org/10.34171/mjiri.33.8>

56. Bellizzi V, De Nicola L, Minutolo R, et al (1999) Effects of water hardness on urinary risk factors for kidney stones in patients with idiopathic nephrolithiasis. Nephron 81 Suppl 1:66-70 <https://doi.org/10.1159/000046301>

57. Berkemeyer S, Bhargava A, Bhargava U (2007) Urinary phosphorus rather than urinary calcium possibly increases renal stone formation in a sample of Asian Indian, male stone-formers. The British journal of nutrition 98:1224-1228 <https://doi.org/10.1017/s0007114507778686>

58. Bobulescu IA, Maalouf NM, Capolongo G, et al (2013) Renal ammonium excretion after an acute acid load: blunted response in uric acid stone formers but not in patients with type 2 diabetes. American journal of physiology Renal physiology 305:F1498-1503 <https://doi.org/10.1152/ajprenal.00374.2013>

59. Bobulescu IA, Park SK, Xu LHR, et al (2019) Net Acid Excretion and Urinary Organic Anions in Idiopathic Uric Acid Nephrolithiasis. Clinical journal of the American Society of Nephrology : CJASN 14:411-420 <https://doi.org/10.2215/cjn.10420818>

60. Burtis WJ, Gay L, Insogna KL, et al (1994) Dietary hypercalciuria in patients with calcium oxalate kidney stones. The American journal of clinical nutrition 60:424-429

61. Caudarella R, Rizzoli E, Buffa A, et al (1998) Comparative study of the influence of 3 types of mineral water in patients with idiopathic calcium lithiasis. The Journal of urology 159:658-663

62. Dai M, Zhao A, Liu A, et al (2013) Dietary factors and risk of kidney stone: a case-control study in southern China. Journal of renal nutrition : the official journal of the Council on Renal Nutrition of the National Kidney Foundation 23:e21-28 <https://doi.org/10.1053/j.jrn.2012.04.003>

63. de OGMC, Martini LA, Baxmann AC, et al (2003) Effects of an oxalate load on urinary oxalate excretion in calcium stone formers. Journal of renal nutrition : the official journal of the Council on Renal Nutrition of the National Kidney Foundation 13:39-46 <https://doi.org/10.1053/jren.2003.50002>

64. De SK, Liu X, Monga M (2014) Changing trends in the American diet and the rising prevalence of kidney stones. Urology 84:1030-1033 <https://doi.org/10.1016/j.urology.2014.06.037>

65. Domrongkitchaiporn S, Stitchantrakul W, Kochakarn W (2006) Causes of hypocitraturia in recurrent calcium stone formers: focusing on urinary potassium excretion. American journal of kidney diseases : the official journal of the National Kidney Foundation 48:546-554 <https://doi.org/10.1053/j.ajkd.2006.06.008>

66. Dongre AR, Rajalakshmi M, Deshmukh PR, et al (2017) Risk Factors for Kidney Stones in Rural Puducherry: Case-Control Study. Journal of clinical and diagnostic research : JCDR 11:Lc01-lc05 <https://doi.org/10.7860/jcdr/2017/29465.10561>

67. Dussol B, Iovanna C, Rotily M, et al (2008) A randomized trial of low-animal-protein or high-fiber diets for secondary prevention of calcium nephrolithiasis. Nephron Clinical practice 110:c185-194 <https://doi.org/10.1159/000167271>

68. Fatiha L, Fouzia RF, Ali A, et al (2009) Correlations between the composition of Moroccan urinary stones and the risk factors (food habit). Pakistan Journal of Nutrition 8:977-982

69. Fellström B, Danielson BG, KarlstrÖM B, et al (1989) Dietary habits in renal stone patients compared with healthy subjects. British journal of urology 63:575-580

70. Gambaro G, Bertaglia G, Inelmen EM, et al (1993) Diet and nephrolithiasis: Study in an obese population. Nutrition Research 13:535-540

71. Gasińska A, Gajewska D (2007) Tea and coffee as the main sources of oxalate in diets of patients with kidney oxalate stones. Roczniki Panstwowego Zakladu Higieny 58:61-67

72. Goldfarb DS, Fischer ME, Keich Y, et al (2005) A twin study of genetic and dietary influences on nephrolithiasis: a report from the Vietnam Era Twin (VET) Registry. Kidney international 67:1053-1061 <https://doi.org/10.1111/j.1523-1755.2005.00170.x>

73. Gordiano EA, Tondin LM, Miranda RC, et al (2014) Evaluation of food intake and excretion of metabolites in nephrolithiasis. Jornal brasileiro de nefrologia : 'orgao oficial de Sociedades Brasileira e Latino-Americana de Nefrologia 36:437-445 <https://doi.org/10.5935/0101-2800.20140063>

74. Griffith HM, O'Shea B, Maguire M, et al (1986) A case-control study of dietary intake of renal stone patients. II. Urine biochemistry and stone analysis. Urological research 14:75-82 <https://doi.org/10.1007/bf00257892>

75. Guerra A, Folesani G, Mena P, et al (2014) Hippuric acid in 24 h urine collections as a biomarker of fruits and vegetables intake in kidney stone formers. International journal of food sciences and nutrition 65:1033-1038 <https://doi.org/10.3109/09637486.2014.950210>

76. Guerra A, Ticinesi A, Allegri F, et al (2019) Insights about urinary hippuric and citric acid as biomarkers of fruit and vegetable intake in patients with kidney stones: The role of age and sex. Nutrition (Burbank, Los Angeles County, Calif) 59:83-89 <https://doi.org/10.1016/j.nut.2018.07.112>

77. Hamid R, Robertson WG, Woodhouse CR (2008) Comparison of biochemistry and diet in patients with enterocystoplasty who do and do not form stones. BJU international 101:1427-1432 <https://doi.org/10.1111/j.1464-410X.2008.07492.x>

78. Hassapidou MN, Paraskevopoulos ST, Karakoltsidis PA, et al (1999) Dietary habits of patients with renal stone disease in Greece. Journal of Human Nutrition and Dietetics 12:47-51

79. Heningburg AM, Mohapatra A, Potretzke AM, et al (2016) Electronic nutritional intake assessment in patients with urolithiasis: A decision impact analysis. Investigative and clinical urology 57:196-201 <https://doi.org/10.4111/icu.2016.57.3.196>

80. Hirvonen T, Pietinen P, Virtanen M, et al (1999) Nutrient intake and use of beverages and the risk of kidney stones among male smokers. American journal of epidemiology 150:187-194 <https://doi.org/10.1093/oxfordjournals.aje.a009979>

81. Hsu TC, Chen J, Huang HS, et al (2002) Association of changes in the pattern of urinary calculi in Taiwanese with diet habit change between 1956 and 1999. Journal of the Formosan Medical Association = Taiwan yi zhi 101:5-10

82. Icer MA, Gezmen-Karadag M (2019) The potential effects of dietary food and beverage intakes on the risk of kidney stone formation. Revista de Nutrição 32

83. Iguchi M, Kataoka K, Kohri K, et al (1984) Nutritional risk factors in calcium stone disease in Japan. Urologia internationalis 39:32-35 <https://doi.org/10.1159/000280940>

84. Iguchi M, Umekawa T, Ishikawa Y, et al (1990) Dietary intake and habits of Japanese renal stone patients. The Journal of urology 143:1093-1095

85. Jabbar F, Asif M, Dutani H, et al (2015) Assessment of the role of general, biochemical and family history characteristics in kidney stone formation. Saudi journal of biological sciences 22:65-68 <https://doi.org/10.1016/j.sjbs.2014.06.002>

86. Karagiannis A, Skolarikos A, Alexandrescu E, et al (2017) Epidemiologic study of urolithiasis in seven countries of South-Eastern Europe: S.E.G.U.R. 1 study. Archivio italiano di urologia, andrologia : organo ufficiale [di] Societa italiana di ecografia urologica e nefrologica 89:173-177 <https://doi.org/10.4081/aiua.2017.3.173>

87. Karagülle O, Smorag U, Candir F, et al (2007) Clinical study on the effect of mineral waters containing bicarbonate on the risk of urinary stone formation in patients with multiple episodes of CaOx-urolithiasis. World journal of urology 25:315-323 <https://doi.org/10.1007/s00345-007-0144-0>

88. Kaufman DW, Kelly JP, Curhan GC, et al (2008) Oxalobacter formigenes may reduce the risk of calcium oxalate kidney stones. Journal of the American Society of Nephrology : JASN 19:1197-1203 <https://doi.org/10.1681/asn.2007101058>

89. Khambati A, Matulewicz RS, Perry KT, et al (2017) Factors Associated with Compliance to Increased Fluid Intake and Urine Volume Following Dietary Counseling in First-Time Kidney Stone Patients. Journal of endourology 31:605-610 <https://doi.org/10.1089/end.2016.0836>

90. Kıraç M, Küpeli B, Irkilata L, et al (2013) Effects of dietary interventions on 24-hour urine parameters in patients with idiopathic recurrent calcium oxalate stones. The Kaohsiung journal of medical sciences 29:88-92 <https://doi.org/10.1016/j.kjms.2012.08.015>

91. Kocvara R, Plasgura P, Petrík A, et al (1999) A prospective study of nonmedical prophylaxis after a first kidney stone. BJU international 84:393-398 <https://doi.org/10.1046/j.1464-410x.1999.00216.x>

92. Krieger JN, Kronmal RA, Coxon V, et al (1996) Dietary and behavioral risk factors for urolithiasis: potential implications for prevention. American journal of kidney diseases : the official journal of the National Kidney Foundation 28:195-201 <https://doi.org/10.1016/s0272-6386(96)90301-7>

93. Kumar MKR (2015) Weight Gain and the Risk of Kidney Stones. Journal of Pharmaceutical Sciences and Research 7:776

94. Lange JN, Easter L, Amoroso R, et al (2013) Internet program for facilitating dietary modifications limiting kidney stone risk. The Canadian journal of urology 20:6922-6926

95. Leonetti F, Dussol B, Berthezene P, et al (1998) Dietary and urinary risk factors for stones in idiopathic calcium stone formers compared with healthy subjects. Nephrology, dialysis, transplantation : official publication of the European Dialysis and Transplant Association - European Renal Association 13:617-622 <https://doi.org/10.1093/ndt/13.3.617>

96. Lieske JC, Tremaine WJ, De Simone C, et al (2010) Diet, but not oral probiotics, effectively reduces urinary oxalate excretion and calcium oxalate supersaturation. Kidney international 78:1178-1185 <https://doi.org/10.1038/ki.2010.310>

97. Lingeman JE, Pareek G, Easter L, et al (2019) ALLN-177, oral enzyme therapy for hyperoxaluria. International urology and nephrology 51:601-608 <https://doi.org/10.1007/s11255-019-02098-1>

98. Littlejohns TJ, Neal NL, Bradbury KE, et al (2020) Fluid Intake and Dietary Factors and the Risk of Incident Kidney Stones in UK Biobank: A Population-based Prospective Cohort Study. European urology focus 6:752-761 <https://doi.org/10.1016/j.euf.2019.05.002>

99. Maalouf NM, Poindexter JR, Adams-Huet B, et al (2019) Increased production and reduced urinary buffering of acid in uric acid stone formers is ameliorated by pioglitazone. Kidney international 95:1262-1268 <https://doi.org/10.1016/j.kint.2018.11.024>

100. Marić I, Kizivat T, Smolić M, et al (2019) LIFESTYLE RISK FACTORS AND BONE MASS IN RECURRENT STONE-FORMING PATIENTS: A CROSS-SECTIONAL STUDY IN 144 SUBJECTS. Acta clinica Croatica 58:439-445 <https://doi.org/10.20471/acc.2019.58.03.06>

101. Martini LA, Cuppari L, Cunha MA, et al (1998) Potassium and sodium intake and excretion in calcium stone forming patients. Journal of renal nutrition : the official journal of the Council on Renal Nutrition of the National Kidney Foundation 8:127-131 <https://doi.org/10.1016/s1051-2276(98)90003-6>

102. Masai M, Ito H, Kotake T (1995) Effect of dietary intake on urinary oxalate excretion in calcium renal stone formers. British journal of urology 76:692-696

103. Massey LK, Kynast-Gales SA (1998) Substituting milk for apple juice does not increase kidney stone risk in most normocalciuric adults who form calcium oxalate stones. Journal of the American Dietetic Association 98:303-308 <https://doi.org/10.1016/s0002-8223(98)00071-6>

104. Melo TL, Esper PLG, Zambrano LI, et al (2020) Expression of vitamin D receptor, CYP27B1 and CYP24A1 hydroxylases and 1,25-dihydroxyvitamin D(3) levels in stone formers. Urolithiasis 48:19-26 <https://doi.org/10.1007/s00240-019-01163-9>

105. Mente A, Irvine EJ, Honey RJ, et al (2009) Urinary potassium is a clinically useful test to detect a poor quality diet. The Journal of nutrition 139:743-749 <https://doi.org/10.3945/jn.108.098319>

106. Meschi T, Nouvenne A, Ticinesi A, et al (2012) Dietary habits in women with recurrent idiopathic calcium nephrolithiasis. Journal of translational medicine 10:63 <https://doi.org/10.1186/1479-5876-10-63>

107. Messa P, Marangella M, Paganin L, et al (1997) Different dietary calcium intake and relative supersaturation of calcium oxalate in the urine of patients forming renal stones. Clinical Science 93:257-263

108. Miladipour AH, Shakhssalim N, Parvin M, et al (2012) Effect of Ramadan fasting on urinary risk factors for calculus formation. Iranian journal of kidney diseases 6:33-38

109. Nagy EN, Tilinca MC, Iacob A, et al (2017) Study on Chemical Composition of Urinary and Salivary Gland Stones in Relationship with Laboratory Parameters and Lifestyle Habits of Patients with Lithiasis. REVISTA DE CHIMIE 68:680-682

110. Nascimento L, Oliveros FH, Cunningham E (1984) Renal handling of sodium and calcium in hypercalciuria. Clinical pharmacology and therapeutics 35:342-347 <https://doi.org/10.1038/clpt.1984.41>

111. Nishiura JL, Martini LA, Mendonça CO, et al (2002) Effect of calcium intake on urinary oxalate excretion in calcium stone-forming patients. Brazilian journal of medical and biological research = Revista brasileira de pesquisas medicas e biologicas 35:669-675 <https://doi.org/10.1590/s0100-879x2002000600006>

112. Nomura K, Ito H, Masai M, et al (1995) Reduction of urinary stone recurrence by dietary counseling after SWL. Journal of endourology 9:305-312 <https://doi.org/10.1089/end.1995.9.305>

113. Oliveira LM, Hauschild DB, Leite Cde M, et al (2014) Adequate dietary intake and nutritional status in patients with nephrolithiasis: new targets and objectives. Journal of renal nutrition : the official journal of the Council on Renal Nutrition of the National Kidney Foundation 24:417-422 <https://doi.org/10.1053/j.jrn.2014.06.003>

114. Pak CY, Odvina CV, Pearle MS, et al (2005) Effect of dietary modification on urinary stone risk factors. Kidney international 68:2264-2273 <https://doi.org/10.1111/j.1523-1755.2005.00685.x>

115. Pendse AK, Singh PP (1986) The etiology of urolithiasis in Udaipur (western part of India). Urological research 14:59-62 <https://doi.org/10.1007/bf00257889>

116. Pieras E, Costa-Bauzá A, Ramis M, et al (2006) Papillary and nonpapillary calcium oxalate monohydrate renal calculi: comparative study of etiologic factors. TheScientificWorldJournal 6:2411-2419 <https://doi.org/10.1100/tsw.2006.374>

117. Primiano A, Persichilli S, Ferraro PM, et al (2020) A Specific Urinary Amino Acid Profile Characterizes People with Kidney Stones. Disease markers 2020:8848225 <https://doi.org/10.1155/2020/8848225>

118. Rao PN, Gordon C, Davies D, et al (1984) Metabolic response to refined carbohydrates in idiopathic urolithiasis. Urologia internationalis 39:165-169 <https://doi.org/10.1159/000280967>

119. Rao PN, Prendiville V, Buxton A, et al (1982) Dietary management of urinary risk factors in renal stone formers. British journal of urology 54:578-583

120. Rodgers A, Mokoena M, Durbach I, et al (2016) Do teas rich in antioxidants reduce the physicochemical and peroxidative risk factors for calcium oxalate nephrolithiasis in humans? Pilot studies with Rooibos herbal tea and Japanese green tea. Urolithiasis 44:299-310 <https://doi.org/10.1007/s00240-015-0855-4>

121. Rodgers AL (1997) Effect of mineral water containing calcium and magnesium on calcium oxalate urolithiasis risk factors. Urologia internationalis 58:93-99 <https://doi.org/10.1159/000282958>

122. Rodrigues FG, Lima TM, Zambrano L, et al (2020) Dietary pattern analysis among stone formers: resemblance to a DASH-style diet. Jornal brasileiro de nefrologia : 'orgao oficial de Sociedades Brasileira e Latino-Americana de Nefrologia 42:338-348 <https://doi.org/10.1590/2175-8239-jbn-2019-0183>

123. Rotily M, Léonetti F, Iovanna C, et al (2000) Effects of low animal protein or high-fiber diets on urine composition in calcium nephrolithiasis. Kidney international 57:1115-1123 <https://doi.org/10.1046/j.1523-1755.2000.00939.x>

124. Ryu HY, Lee YK, Park J, et al (2018) Dietary risk factors for urolithiasis in Korea: A case-control pilot study. Investigative and clinical urology 59:106-111 <https://doi.org/10.4111/icu.2018.59.2.106>

125. Schwen ZR, Riley JM, Shilo Y, et al (2013) Dietary management of idiopathic hyperoxaluria and the influence of patient characteristics and compliance. Urology 82:1220-1225 <https://doi.org/10.1016/j.urology.2013.08.002>

126. Shafi H, Dorosty Motlagh AR, Bagherniya M, et al (2017) The Association of Household Food Insecurity and the Risk of Calcium Oxalate Stones. Urology journal 14:4094-5000

127. Shavit L, Ferraro PM, Johri N, et al (2015) Effect of being overweight on urinary metabolic risk factors for kidney stone formation. Nephrology, dialysis, transplantation : official publication of the European Dialysis and Transplant Association - European Renal Association 30:607-613 <https://doi.org/10.1093/ndt/gfu350>

128. Siener R, Hoppe B, Löhr P, et al (2018) Metabolic profile and impact of diet in patients with primary hyperoxaluria. International urology and nephrology 50:1583-1589 <https://doi.org/10.1007/s11255-018-1939-1>

129. Siener R, Netzer L, Hesse A (2013) Determinants of brushite stone formation: a case-control study. PloS one 8:e78996 <https://doi.org/10.1371/journal.pone.0078996>

130. Siener R, Petzold J, Bitterlich N, et al (2013) Determinants of urolithiasis in patients with intestinal fat malabsorption. Urology 81:17-24 <https://doi.org/10.1016/j.urology.2012.07.107>

131. Siener R, Schade N, Nicolay C, et al (2005) The efficacy of dietary intervention on urinary risk factors for stone formation in recurrent calcium oxalate stone patients. The Journal of urology 173:1601-1605 <https://doi.org/10.1097/01.ju.0000154626.16349.d3>

132. Soldati L, Bertoli S, Terranegra A, et al (2014) Relevance of Mediterranean diet and glucose metabolism for nephrolithiasis in obese subjects. Journal of translational medicine 12:34 <https://doi.org/10.1186/1479-5876-12-34>

133. Somashekara HM, Urooj A (2015) Nutritional status and dietary habits of subjects with urolithiasis. Current Research in Nutrition and Food Science Journal 3:46-53

134. Stitchantrakul W, Kochakarn W, Ruangraksa C, et al (2007) Urinary risk factors for recurrent calcium stone formation in Thai stone formers. Journal of the Medical Association of Thailand = Chotmaihet thangphaet 90:688-698

135. Stout* T, Lingeman J, Krambeck A, et al (2019) MP12-05 UTILIZATION OF A SMART WATER BOTTLE TO INCREASE FLUID INTAKE IN STONE FORMERS. The Journal of urology 201:e169-e170

136. Sweeney DD, Tomaszewski JJ, Ricchiuti DD, et al (2009) Effect of carbohydrate-electrolyte sports beverages on urinary stone risk factors. The Journal of urology 182:992-997 <https://doi.org/10.1016/j.juro.2009.05.020>

137. Tessaro CZW, Ramos CI, Heilberg IP (2018) Influence of nutritional status, laboratory parameters and dietary patterns upon urinary acid excretion in calcium stone formers. Jornal brasileiro de nefrologia : 'orgao oficial de Sociedades Brasileira e Latino-Americana de Nefrologia 40:35-43 <https://doi.org/10.1590/2175-8239-jbn-3814>

138. Thomas LD, Elinder CG, Tiselius HG, et al (2013) Dietary cadmium exposure and kidney stone incidence: a population-based prospective cohort study of men & women. Environment international 59:148-151 <https://doi.org/10.1016/j.envint.2013.06.008>

139. Ticinesi A, Milani C, Guerra A, et al (2018) Understanding the gut-kidney axis in nephrolithiasis: an analysis of the gut microbiota composition and functionality of stone formers. Gut 67:2097-2106 <https://doi.org/10.1136/gutjnl-2017-315734>

140. Toren PJ, Norman RW (2005) Is 24-hour urinary calcium a surrogate marker for dietary calcium intake? Urology 65:459-462 <https://doi.org/10.1016/j.urology.2004.10.025>

141. Tosukhowong P, Boonla C, Ratchanon S, et al (2007) Crystalline composition and etiologic factors of kidney stone in Thailand: update 2007.

142. Trinchieri A, Lizzano R, Marchesotti F, et al (2006) Effect of potential renal acid load of foods on urinary citrate excretion in calcium renal stone formers. Urological research 34:1-7 <https://doi.org/10.1007/s00240-005-0001-9>

143. Trinchieri A, Maletta A, Lizzano R, et al (2013) Potential renal acid load and the risk of renal stone formation in a case-control study. European journal of clinical nutrition 67:1077-1080 <https://doi.org/10.1038/ejcn.2013.155>

144. Trinchieri A, Mandressi A, Luongo P, et al (1991) The influence of diet on urinary risk factors for stones in healthy subjects and idiopathic renal calcium stone formers. British journal of urology 67:230-236

145. Trinchieri A, Nespoli R, Ostini F, et al (1998) A study of dietary calcium and other nutrients in idiopathic renal calcium stone formers with low bone mineral content. The Journal of urology 159:654-657

146. Trinchieri A, Zanetti G, Currò A, et al (2001) Effect of potential renal acid load of foods on calcium metabolism of renal calcium stone formers. European urology 39 Suppl 2:33-36; discussion 36-37 <https://doi.org/10.1159/000052556>

147. Turney BW, Appleby PN, Reynard JM, et al (2014) Diet and risk of kidney stones in the Oxford cohort of the European Prospective Investigation into Cancer and Nutrition (EPIC). European journal of epidemiology 29:363-369 <https://doi.org/10.1007/s10654-014-9904-5>

148. Vezzoli G, Dogliotti E, Terranegra A, et al (2015) Dietary style and acid load in an Italian population of calcium kidney stone formers. Nutrition, metabolism, and cardiovascular diseases : NMCD 25:588-593 <https://doi.org/10.1016/j.numecd.2015.03.005>

149. Wollin DA, Davis LG, Winship BB, et al (2020) Assessment of conservative dietary management as a method for normalization of 24-h urine pH in stone formers. Urolithiasis 48:131-136 <https://doi.org/10.1007/s00240-019-01139-9>

150. Worcester EM, Bergsland KJ, Gillen DL, et al (2020) Evidence for disordered acid-base handling in calcium stone-forming patients. American Journal of Physiology-Renal Physiology 318:F363-F374

151. Yasui T, Okada A, Hamamoto S, et al (2013) The association between the incidence of urolithiasis and nutrition based on Japanese National Health and Nutrition Surveys. Urolithiasis 41:217-224 <https://doi.org/10.1007/s00240-013-0567-6>

152. Zechner O, Latal D, Pflüger H, et al (1981) Nutritional risk factors in urinary stone disease. The Journal of urology 125:51-54

153. Zeng G, Mai Z, Xia S, et al (2017) Prevalence of kidney stones in China: an ultrasonography based cross-sectional study. BJU international 120:109-116 <https://doi.org/10.1111/bju.13828>

154. Zhao A, Dai M, Chen YJ, et al (2015) Risk factors associated with nephrolithiasis: a case-control study in China. Asia-Pacific journal of public health 27:Np414-424 <https://doi.org/10.1177/1010539512445189>

155. Zhuo D, Li M, Cheng L, et al (2019) A Study of Diet and Lifestyle and the Risk of Urolithiasis in 1,519 Patients in Southern China. Medical science monitor : international medical journal of experimental and clinical research 25:4217-4224 <https://doi.org/10.12659/msm.916703>

156. Duan X, Zhang T, Ou L, et al (2020) (1)H NMR-based metabolomic study of metabolic profiling for the urine of kidney stone patients. Urolithiasis 48:27-35 <https://doi.org/10.1007/s00240-019-01132-2>

157. Borghi L, Schianchi T, Meschi T, et al (2002) Comparison of two diets for the prevention of recurrent stones in idiopathic hypercalciuria. The New England journal of medicine 346:77-84 <https://doi.org/10.1056/NEJMoa010369>

158. Meschi T, Maggiore U, Fiaccadori E, et al (2004) The effect of fruits and vegetables on urinary stone risk factors. Kidney international 66:2402-2410 <https://doi.org/10.1111/j.1523-1755.2004.66029.x>

159. Nouvenne A, Meschi T, Guerra A, et al (2009) Diet to reduce mild hyperoxaluria in patients with idiopathic calcium oxalate stone formation: a pilot study. Urology 73:725-730, 730.e721 <https://doi.org/10.1016/j.urology.2008.11.006>

160. Nouvenne A, Meschi T, Prati B, et al (2010) Effects of a low-salt diet on idiopathic hypercalciuria in calcium-oxalate stone formers: a 3-mo randomized controlled trial. The American journal of clinical nutrition 91:565-570 <https://doi.org/10.3945/ajcn.2009.28614>

161. Nouvenne A, Ticinesi A, Allegri F, et al (2014) Twenty-five years of idiopathic calcium nephrolithiasis: has anything changed? Clinical chemistry and laboratory medicine 52:337-344 <https://doi.org/10.1515/cclm-2013-0618>

162. Hiatt RA, Ettinger B, Caan B, et al (1996) Randomized controlled trial of a low animal protein, high fiber diet in the prevention of recurrent calcium oxalate kidney stones. American journal of epidemiology 144:25-33 <https://doi.org/10.1093/oxfordjournals.aje.a008851>
