# Supplementary material for: Methods for the dietary assessment of adult kidney stone formers: a scoping review
Source: J Nephrol. 2022 Feb 15;35(3):821–30. doi: 10.1007/s40620-022-01259-3 (PMC8995246; doi:10.1007/s40620-022-01259-3)
Supplement: Supplementary file 3 — Supplementary file3 (PDF 113 kb) [file 40620_2022_1259_MOESM3_ESM.pdf]

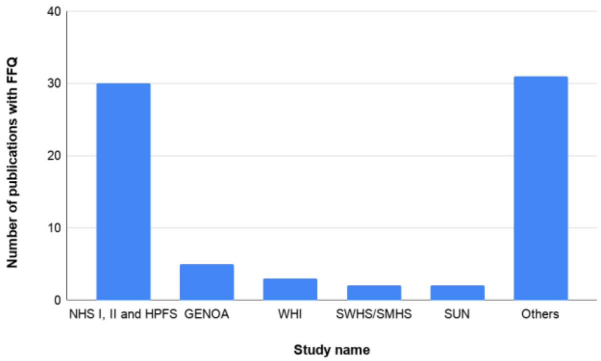

Fig.7 Number of publications per study with a FFQ (n=73)

NHS I and II: Nurses' Health Study I and II
HPFS: Health Professionals Follow-Up Study
GENOA: The Genetic Epidemiology Network of Arteriopathy cohort
WHI: The Women's Health Initiative Observational Study
SWHS/SMHS: Shanghai Women's Health Study and Shanghai Men's Health Study
SUN: The Seguimiento Universidad de Navarra
